# Casein-Conjugated Gold Nanoparticles for Amperometric Detection of *Leishmania infantum*

**DOI:** 10.3390/bios9020068

**Published:** 2019-05-27

**Authors:** Mohamed Fethi Diouani, Oussama Ouerghi, Kamel Belgacem, Maher Sayhi, Radu Ionescu, Dhafer Laouini

**Affiliations:** 1 Institut Pasteur de Tunis, LR11IPT03, Laboratory of Epidemiology and Veterinary Microbiology (LEMV), Tunis-Belvédère 1002, Tunisia; fethi.diouani@pasteur.tn (M.F.D.); kamelbelgacem85@gmail.com (K.B.); sayhimaher@ymail.com (M.S.); 2 Prince Sattam Bin Abdulaziz University, Al Kahrj 11942, Saudi Arabia; 3 Université Tunis El Manar, Tunis 1068, Tunisia; dhafer.laouini@pasteur.tn; 4 The Ångström Laboratory, Division of Solid State Physics, Department of Engineering Sciences, Uppsala University, 75121 Uppsala, Sweden; radu.ionescu@angstrom.uu.se; 5 Institut Pasteur de Tunis, LR11IPT02, Laboratory of Transmission, Control and Immunobiology of Infections (LTCII), Tunis-Belvédère 1002, Tunisia

**Keywords:** *Leishmania infantum*, gold nanoparticles, casein, chronoamperometry, GP63

## Abstract

Sensitive and reliable approaches targeting the detection of *Leishmania* are critical for effective early diagnosis and treatment of leishmaniasis. In this frame, this paper describes a rapid quantification assay to detect *Leishmania* parasites based on the combination of the electrocatalytic ability of gold nanoparticles (AuNPs) to act as a catalyst for the hydrogen formation reaction along with the specificity of the interaction between casein and the major surface protease of the *Leishmania* parasite, GP63. First, pure and casein-modified AuNPs were prepared and characterized by scanning electron microscopy and ultraviolet–visible spectroscopy. Then, casein-conjugated AuNPs were incubated with *Leishsmania* parasites in solution; the formed complex was collected by centrifugation, treated by acidic solution, and the pelleted AuNPs were placed on screen-printed carbon electrodes (SPCEs) and chronoamperometric measurements were carried out. Our results suggest that it is possible to detect *Leishmania* parasites, with a limit less than 1 parasite/mL. A linear response over a wide concentration interval, ranging from 2 × 10^−2^ to 2 × 10^5^ parasites/mL, was achieved. Additionally, a pretreatment of *Leishmania* parasites with Amphotericin B, diminished their interaction with casein. This findings and methodology are very useful for drug efficacy assessment.

## 1. Introduction

*Leishmania* (*L.*) is an intracellular protozoan parasite transmitted by phlebotomine sandflies. It causes a wide range of human diseases, ranging from cutaneous lesions to visceral dissemination. As reported by the World Health Organization (WHO), leishmaniasis is one of the six most important diseases worldwide, putting at risk over 350 million people and causing ~1.6 million new cases annually in 98 countries, including developed and developing countries alike [1,2]. However, the majority of cases (more than 90%) occur in low-income countries suffering from the lack of necessary health resources for effective diagnosis and control of the disease [2]. In the Mediterranean region, including Tunisia, visceral leishmaniasis (VL) is mainly due to *L. infantum* species, with dogs as the major natural reservoir of the parasite and playing a crucial role in the transmission and spread of the disease [3]. Conventional golden standard assays for leishmaniasis diagnosis include historically the microscopic approach, based on the observation or isolation of the infectious agent for in-lab confirmation of the infection; serology-based assays, based on the detection of specific antibodies against leishmaniosis infection; and molecular-based approaches, based on nucleic acids and often using polymerase chain reaction (PCR) techniques [4,5,6,7]. Despite their sensitivity, these techniques require specific and costly equipment, highly-skilled staff, and long turnaround times. Due to the limitations of these conventional detection methods, it is necessary to develop reliable diagnostic assays. The advantages acquired from nanotechnology and its application for the development of biosensors can be used friendly without any needs for qualified technicians. These devices could significantly support the growing efforts devoted to decrease the impact of the disease on public health for humans and animals alike.

Basically, the life cycle of *Leishmania* involves two phases. In the first phase, motile and flagellated promastigotes reside in the midgut of phlebotomine sandflies. Promastigotes are then injected into a mammalian host by the vector (phlebotomine sandflies) during an accidental feeding. During the second phase, promastigotes are internalized in the macrophages of the mammalian host, where they transform into nonmotile amastigotes [8].

*Leishmania* species express an abundant surface glycoprotein, known as leishmanolysin and referred to as GP63 [9]. The latter is assumed to be a ligand involved in the interactions between the parasite and the host’s immune system, including components of the complement system and the macrophage surface [10]. Thus, GP63 weakens the activity of macrophages tolerating intracellular invasion, allowing the spread and the survival of the parasite. GP63/leishmanolysin, also known as the major surface protease (MSP), is a zinc-dependent metalloprotease overexpressed at the surface of the parasite, or directly secreted by Leishmania. This enzyme is expressed predominantly in the promastigote stage, whereas it drops to low levels as the parasite transmutes to its amastigote form [11]. Another important aspect of GP63 is its ability to degrade various substrates, such as casein, hemoglobin, gelatin, fibrinogen, and albumin [12]. A previous study reported casein as the best substrate for detecting GP63-like proteins in *Herpetomonas megaseliae* parasites [13].

Caseins, the most abundant proteins in milk, are amphiphilic block copolymers futured by a large number of hydrophobic or hydrophilic amino acid residues. Thanks to their amphiphilicity in aqueous solution, they experience a high ability to self-assemble into stable micelles [14,15]. Stability against aggregation of casein micelles, mainly controlled by the steric repulsion between the polypeptide brushes, makes them useful for the production of stabilized gold nanoparticles (AuNPs) in aqueous solutions [16]. However, in addition to offering high stability for the system, the conjugation of caseins with AuNPs also provides these nanoparticles with the biocompatible functionalities that are often required for many biological applications [17,18]. Previous work reported a casein-based AuNP assay to assess the *E. coli*–casein interaction by monitoring catalytic current associated with the hydrogen formation reaction performed on screen-printed carbon electrodes [19].

Among the wide variety of nanoparticles, AuNPs have been the focus of many research studies due to their exceptional physicochemical properties, i.e., structural, electric, optic, magnetic, and catalytic, which make them very attractive for versatile bioassays and biosensors applications [20,21,22,23]. Particularly, the catalytic activity of AuNPs for the hydrogen reaction formation has paved the way for the building of highly sensitive sensors with large sensing properties [24,25,26,27,28].

Conceptually, the AuNPs, deposited on the surface of a screen-printed carbon electrode, release a large number of Au^3+^ ions from each gold nanoparticle. These ions provide free electroactive sites to the protons (H^+^) present in the acidic medium (HCl). The protons were then catalytically reduced to hydrogen under an adequate potential, producing a shift in the cathodic current. For biodetection applications, the target biomolecule reacts with its specific probes that were already labeled by gold nanoparticles. The formed complex, when treated by an acid, releases gold nanoparticle that can be quantified by chronoamperometry, which in turn allows quantification of target biomolecules. In other words, target biomolecules are indirectly quantified via the quantification of the gold nanoparticles anchored to the probes.

Encouraged by the above-mentioned studies, the present work is based on the dual use of AuNPs as nanocarriers and as electrocatalytic labels with the potentiality of casein to act as a substrate for the GP63 protease. We used these advantages to design a sensing assay for *Leishmania* parasites detection. In fact, casein-conjugated AuNPs were incubated with *Leishmania* parasites at different concentrations. The formed complexes were collected by centrifugation, treated by acidic solution then the collected AuNPs were placed on the working electrode surface of screen-printed carbon electrodes (SPCEs) and chronoamperometric measurements were carried out. Principally, we processed by monitoring the cathodic current associated with the reduction of protons (H^+^) to hydrogen, in acidic medium, amplified by the catalytic effect of AuNPs when a suitable potential is imposed to the working electrode. The recorded current is correlated with the concentration of AuNPs quantified inside the test medium, and in turn quantifies *Leishmania* parasites. Such a method promises substantial potential for use in human and veterinary medicine applications, particularly in limited-resources settings.

## 2. Materials and Methods

### 2.1. Reagents and Apparatuses

Caseino-glycopeptide (CGP), hydrogen tetrachloroaurate (III) trihydrate (HAuCl_4__3H_2_O, 99.9%), trisodium citrate (Na_3_C_6_H_5_O_7_.2H_2_O), Amphotericin B (AmB), RPMI-1640 medium, gentamicin, and fetal bovine serum were purchased from Sigma-Aldrich (Saint-Quentin, France). Potassium dihydrogen phosphate (KH_2_PO_4_) and dipotassium hydrogen phosphate (K_2_HPO_4_) reagents were purchased from Fluka (Saint-Quentin, France) and were used to prepare phosphate buffer solutions (0.01 M PBS, pH 6.8 and pH 7.4).

The *L. infantum* parasites (IPT1 strain) were routinely maintained in culture in our laboratory and were collected from our previous epidemiological investigation on canine leishmaniasis in endemic area of Tunisia [29].

Cyclic voltammetry and chronoamerometry experiments were performed on a computerized PGZ 301 Voltalab 40 potentiostat purchased from radiometer analytical instrument S.A. (Hach Lange France, Marnes-La-Vallée, France) Screen-printed carbon electrodes (SPCEs, DRP-110), as well as their specific connector to the potensiostat, were acquired from DropSens (LIanera, Asturias, Spain). The SPCEs consist of a three-electrode test cell, printed on ceramic substrates (33 × 10 × 0.5 mm) including a circular-shaped working electrode with a diameter of 4mm, a counter and a pseudo reference electrode made of silver. Working and counter electrodes are both made of carbon ink. A ring-shaped insulating layer around the working electrode with a capacity of 50 μL was incorporated into the SPCES to form the reservoir of the test cell.

Optical characterization of the as-prepared AuNPs was performed by a UV–Vis spectrophotometer (Single Beam LI-295, Lasany, India), while morphological characterization was carried out by a transmission electron microscope (TEM) JEM-1011 from Jeol Ltd., (Tokyo, Japan) operating under high vacuum conditions and an accelerating voltage of 150 kV.

X-ray photoelectron spectroscopy (XPS) analysis of casein-conjugated AuNPs was carried out using a K-Alpha XPS system (Thermo Fisher Scientific, Waltham, MA, USA) that is equipped with a microfocused monochromatic Al Ka X-ray source (1486.6 eV).

### 2.2. Leishmania Parasite Culture

*L. infantum* promastigotes were cultured in RPMI-1640 medium, purchased from Sigma-Aldrich, supplemented with 10% heat-inactivated fetal bovine serum (FBS) and 1% gentamicin (50 mg/mL) at 23 °C. Cultures were seeded by inoculation with early stationary phase promastigotes at starting density of 1 × 10^6^ parasites/mL. Neubauer brightline hemocytometer was used to count the parasites daily.

### 2.3. AuNPs Preparation and Functionalization

AuNPs were prepared as described in our previous work [30] via the reduction reaction of a tetrachloroauric acid with trisodium citrate, following a procedure initially reported by Turkevich with few modifications [31]. In brief, a volume of 200 mL from a 0.01% *w*/*v* HAuCl_4_ solution was heated until boiling while stirring continuously. Then, 5 mL of a 1% *w*/*v* trisodium citrate solution was supplemented rapidly to the latter prepared solution. During this process, the color of the solution changes from yellow to deep red. Finally, the resultant solution was left cooling with stirring, then stored in dark.

The casein–AuNPs conjugates were prepared as previously described by Espinoza-Castañeda [19], who described the optimum conditions to avoid agglomeration caused by casein micelles and the encapsulation of the AuNPs by micelles. Typically, a casein solution of a concentration of 0.1 mg/mL together with a casein/AuNPs concentration ratio of 3:1 are appropriate. The incubation of casein with AuNPs solutions was accomplished by gentle stirring, for 30 min at room temperature. Casein excess was discarded by centrifugation of the former solution at 14000 rpm. The collected pellet was diluted in 0.01 M PBS, at pH 6.8, and the obtained solution was stored in dark at 4 °C. Spectrophotometric analyses were performed by loading 1 mL suspension of the AuNPs and the casein@AuNPs conjugates, separately in Quartz cells and scanning the wavelength from 450 nm to 650 nm.

### 2.4. Incubation of the Parasites with casein@AuNPs and Leishmania Quantification

**Figure 1:**
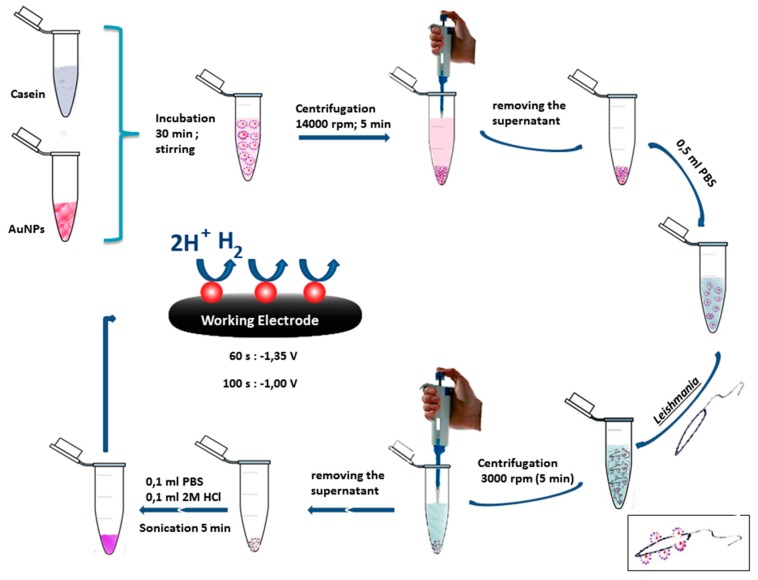
Schematic illustration of the chronoamperometric detection principle of *Leishmania infantum* parasites through hydrogen evolution reaction catalyzed by gold nanoparticles.

Quantitative analyses of *Leishmania* parasites were performed through a chronoamperometric approach and followed a previously optimized procedure [19]. Such analytical procedure is schematically depicted in Figure 1. Briefly, 500 μL casein–AuNPs were incubated with 500 μL of different concentrations of *Leishmania* (from 2 × 10^−2^ to 2 × 10^6^ parasites/mL) for 30 min at room temperature under gentle stirring. At this stage, the AuNPs are attached to *Leishmania* parasites via casein-GP63 interactions. The obtained solution was then centrifuged at 3000 rpm to eliminate the unbounded AuNPs. After removing the supernatant, the collected pellet, containing the AuNPs–casein–*Leishmania* complex, was reinjected in 100 μL of 0.01 M PBS at pH 7.4. Chronoamperograms were achieved by putting down a mixture containing 25 μL of the casein–AuNPs–*Leishmania* complex with 25 μL of 2 M HCl onto the working electrode. Oxidative pretreatment of AuNPs was initially accomplished by application of potential of +1.35 V for 60 s. Next, a potential of −1 V was applied to the working electrode for 100 sec and the generated current was recorded. Subsequently, the hydrogen formation reaction was investigated through the change in the current arising during the catalytic process using a chronoamperometric approach.

### 2.5. Electrochemical Assessment of the Effect of Amphotericin B Pretreatment on Leishmania-casein Interaction

To evaluate the effect of Amphotericin B (AmB) on casein–*Leishmania* interaction, chronoamperometric responses were recorded at different incubation times of the parasite using a fixed concentration of the antibiotic. *Leishmania* parasites (10^4^ /mL) were mixed with 0.3 μg/mL of AmB for 10, 30, 120, 240, and 360 min at 37 °C. Then, casein–AuNPs were added to the mixture for 30 min at room temperature with gentle stirring. Pellets from the reaction mixtures were then collected by centrifugation at 3000 rpm for 10 min, and resuspended in PBS/HCl. Finally, chronoamperometric responses were recorded as described above.

## 3. Results and Discussion

### 3.1. Characterization of Pure and casein-Capped AuNPs

Owing to their extraordinary electrocatalytic activity, AuNPs have been widely exploited as labels for various biorecognition and biosensing processes [32,33]. However, their catalytic performance was found to depend significantly on the particle size [30]. Moreover, the grain size and morphology of the nanoparticles were controlled by the synthesis method conditions [34]. Besides, casein molecules were reported to bind to AuNPs surface, which subsequently led to the formation of casein–AuNP conjugates [16]. Herein, the synthetized AuNPs as well as the casein–AuNPs conjugates were characterized using TEM, UV-Vis and XPS.

**Figure 2:**
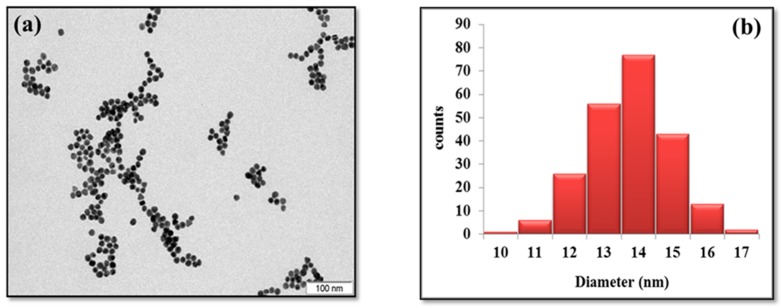
(**a**) TEM image of the prepared AuNPs; (**b**) AuNPs size histogram distribution.

As shown in Figure 2a, the TEM image of the bare AuNPs shows that these colloidal nanoparticles are well dispersed and roughly spherical having an average diameter of ~14 nm determined from the size distribution histogram corresponding to the analysis of the above-mentioned image (Figure 2b). Furthermore, as the electrocatalytic activity of AuNPs is highly affected by their size, sufficiently narrowed size distribution is required to achieve reproducible catalytic effects. For this reason, each experiment in this study was conducted using AuNPs extracted from the same batch to reduce the unavoidable dispersion in the standard deviation of the obtained results.

**Figure 3:**
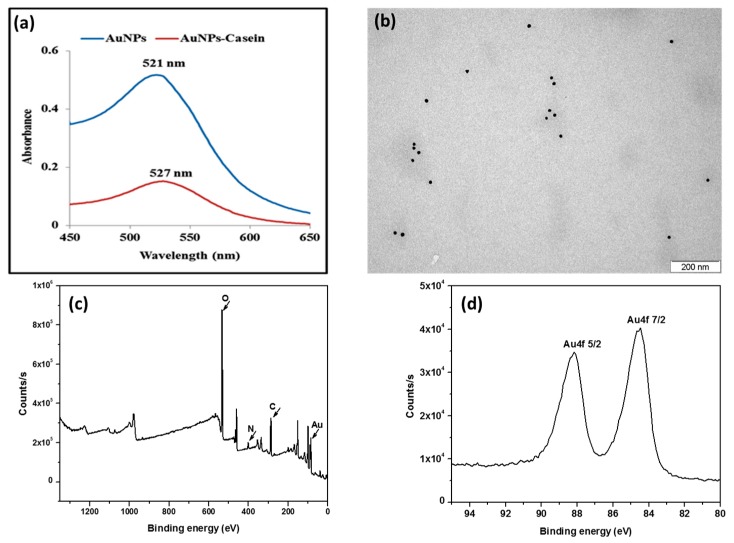
(**a**) UV–visible spectra of bare AuNPs and the casein–AuNPs conjugates; (**b**) TEM image of the casein–AuNPs; (**c**) The whole XPS spectrum of casein@AuNPs; (**d**) XPS spectrum showing the two peaks of the Au4f spin–orbit doublet binding energy.

Besides, the UV–visible absorption spectrum revealed that the maximum absorption shifted from 521 nm for the pure AuNPs to 527 nm after their conjugation with casein (Figure 3a). Such an observed shift to longer wavelengths along with the broadening of the surface-plasmon resonance (SPR) peak could be ascribed to the alterations of the local dielectric constant around the AuNPs because of their conjugation with casein. Moreover, TEM image of the casein–AuNPs conjugates revealed that their size is slightly higher (18 ± 2 nm) with respect to the size of the unmodified AuNPs. This result is consistent with the observed UV-Vis shift and suggests the efficient coating of the AuNPs by casein.

Surface chemical compositions of the casein–AuNPS conjugates were further evaluated by X-ray photoelectron spectroscopy (XPS). The XPS spectrum of casein–AuNPs deposited on a silicon oxide substrate was shown in Figure 3c. Results reveal the presence of gold, carbon, nitrogen and oxygen, indicating that the AuNPs were successfully capped by casein, which is in agreement with the UV–Vis and the TEM results.

Furthermore, Figure 3d shows the XPS spectra of the Au4f doublet (4f_7/2_ and 4f_5/2_) for AuNPs capped with casein. The Au4f_7/2_ and Au4f_5/2_ peaks appeared at 84.6 and 88.3 eV, respectively, which are the typically characteristic of the pure metallic Au^0^ [35,36], confirming the formation of AuNPs.

### 3.2. Electrocatalytic Activity of AuNPs Towards Hydrogen Ions Reduction

Even though gold is an inert metal, the high surface area to volume ratio, surface properties [37,38], and nanoscale dimensions [39] enhance considerably the catalytic activity of the derivative AuNPs, resulting, in turn, to amplified electrical signals [40,41]. The electrocatalytic activity of AuNPs towards the proton reduction was initially investigated by cyclic voltammetry. Indeed, 25 μL of a 2 M HCl was added to 25 μL AuNPs solutions of various concentrations. The derivative mixture was deposited on the working electrode of the SPCEs electrodes. Cyclic voltammetry measurements were then performed by scanning the potential from +1.35 to −1.40 V at a scan rate of 50 mV/s. Likewise, the background signal (blank experiment) was carried out by deposing 50 μL of a 1 M HCl onto the working electrode of the SPCEs.

**Figure 4:**
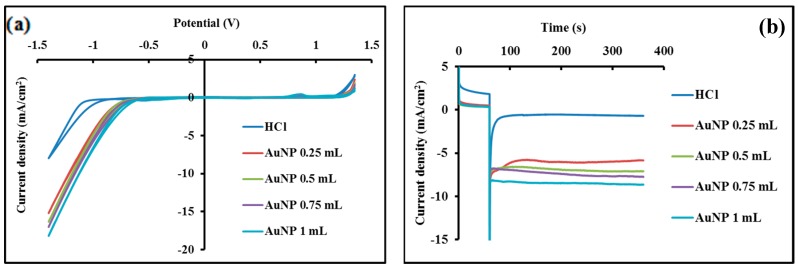
(**a**) Cyclic voltammograms recorded from +1.35 to −1.40 V at a scan rate of 50 mV/s for a 1 M HCl solution and for increasing concentrations of AuNPs in 1 M HCl. (**b**) Chronoamperograms recorded by applying a potential of −1.00 V for 300 s, using a 1 M HCl solution and the same AuNPs concentrations in 1 M HCl.

Data of Figure 4a show that the hydrogen reduction reaction, in the test medium, arises from a potential around −1 V as displayed in the background CV. However, when AuNPs are placed onto the working electrode, the reduction potential for hydrogen ion shifted to less negative potentials (more than 300 mV) as the concentration of AuNPs increases. Likewise, as shown in Figure 4b, because of the catalytic activity of the AuNPs, higher current is observed with increasing AuNP concentration.

Additional investigations concerning the catalytic activity of AuNPs were performed using chronoamperometry approach. The recorded chronoamperograms were accomplished for the as-prepared mixture, and achieved by addition of 25 μL of 2 M HCl to 25 μL AuNP solutions of various concentrations, while imposing a constant potential of +1.35 V on the working electrodes for 1 min. This pretreatment step is crucial for obtaining the greatest catalytic activity and can be explained by the fact that, whenever an oxidative potential is imposed to the working electrode, some atoms from the surface of AuNPs are dissolved in the solution and Au^3+^ ions are formed. These released ions in the solution could enhance the catalytic effect on the hydrogen formation reaction [41]. The cathodic current generated was registered during 300 s, while a constant potential of −1 V was applied to the working electrode. The background signal was performed through the measurement of the cathodic current produced by a 50-μL solution of HCl (1M) placed onto the working electrode surface, while maintaining the same procedure as described above.

The generated current throughout the hydrogen ion reduction process appears to be closely associated with the concentration of AuNPs in the test solution placed onto the working electrode. The amplitude of the recorded current (absolute value), taken at 100 s, was undertaken as the assay’s analytical signal.

### 3.3. Detection of Leishmania Parasites

Usually, the immobilization of immune reagents, such as antibody or antigen on an electrode surface for target analyte detection, is highly required in immunosensor technology. Advantageously, in the present work, labeling of *Leishmania* parasites was achieved in suspension, which allows casein–AuNPs conjugates to interact readily and rapidly with GP63 proteins overexpressed at the surface of the parasite. GP63 proteins were previously used as a recognition element in the design of sensing assays for *Leishmania* parasites [42,43].

Furthermore, it is worth mentioning that the casein–AuNPs–*Leishmania* complex can be destroyed by the acidic process achieved prior to the last detection step. Actually, acidic treatment performed after any biological reaction is a commonly useful approach for the detection of nanoparticles [44]. Biological systems subject to this process are denatured, whereas nanoparticles remain in the detection solution, retain their catalytic ability and their concentration reveals the amount of the target analyte. Following the incubation protocol, *Leishmania* parasites were detected by measurement the chronoamperometric current arising from the catalytic process associated with the hydrogen evolution reaction performed in 1 M HCl solution and catalyzed by AuNPs labels.

**Figure 5:**
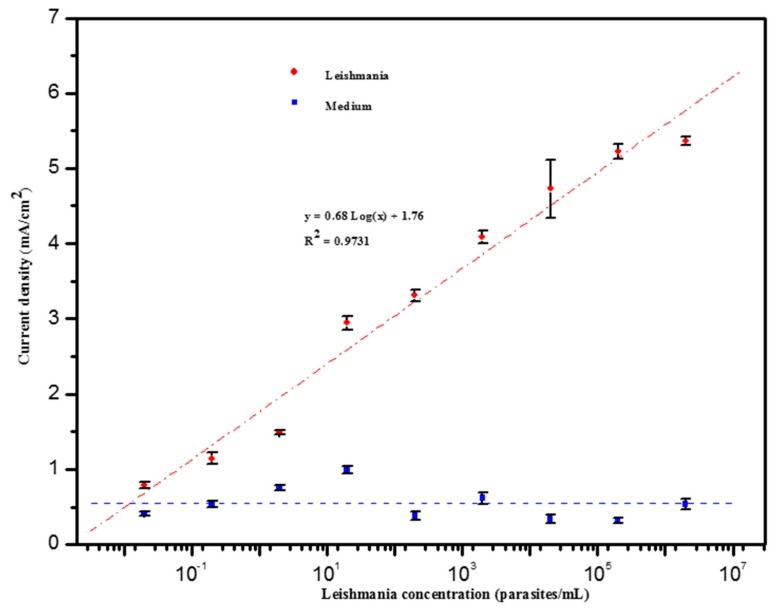
Biosensor response to various concentrations of *Leishmania infantum* parasites (logarithmic range) ranging from 2 × 10^−2^ to 2 × 10^6^ parasites/mL and to various concentrations of parasite-free medium in 1M HCl solution.

Figure 5 shows the evolution of the analytical signal with the concentration of *Leishmania* parasites, over the interval 2 × 10^−2^ and 2 × 10^6^ parasites/mL. A linear relationship was observed between 2 × 10^−2^ and 2 × 10^5^ parasites/mL with a limit of detection (LOD) of about 0.55 parasite/mL and a correlation coefficient of 0.97. LOD was calculated using the following equation, LOD = 3σ/s, where σ is the standard deviation of the blank measurement and s is the slope of the linear part of the calibration curve. The reproducibility of the method shows a relative standard deviation (RSD) around 4%, obtained for 3 repetitive assays. The low limit of detection (less than 1 parasite/mL) is likely due to the fact that GP63 proteins can also be secreted outside by *Leishmania* parasites in the form of microvesicles [45]. Indeed, microvesicle-based secretion appears to be a general mechanism for the secretion of proteins by protozoan parasites [46,47]. It has been previously reported that proteins enclosed inside these microvesicles can be involved in the recognition of parasite infection and pathogen survival is several diseases including malaria and chagas disease. Consequently they could be used for disease treatment [48,49]. On the other hand, these extracellular vesicles are highly immunogenic, and could hence be used as appropriate biomarkers for early detection of parasitic diseases [46].

### 3.4. Effect of the AmB Treatment

**Figure 6:**
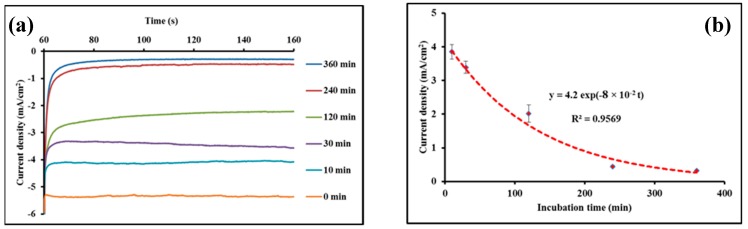
(**a**) Chronoamperometric curves obtained without AmB pretreatment of *Leishmania* parasites (0 min) and with AmB pretreatment for different times (10; 30; 120; 240 and 360 min). (**b**) corresponds to the electrocatalytical signal highlighting the effect of the incubation time of *Leishmania* parasites with AmB on the casein–GP63 interaction. The discontinuous line indicated the fitting of the experimental data with an exponential regression (current density) = 4.2 × exp(−8 × 10^−2^ t), (R^2^ = 0.9569, n = 3).

AmB is considered as one of the most effective drugs for VL treatment, with a cure rate of ~97% and no reported resistance [50,51]. Despite its widespread use, as well as its therapeutic efficiency, there is still controversy about the molecular mechanism of action of AmB. Nevertheless, the widely accepted model is based on the effects of both ergosterol binding and pore formation [52,53]. Previous study reported the inhibition of the growth of *L. donovani* in vitro by application of AmB at a concentration of 0.3 mg/mL [54]. Herein, the influence of incubation time of AmB with *Leishmania* parasites on the subsequent interaction between the latter and casein–AuNPs conjugates was investigated. A solution containing *Leishmania* parasites at a fixed concentration 10^4^ parasites/mL was incubated with a 0.3 mg/mL of AmB for different time intervals, then exposed to casein@AuNPs conjugates. The effect of the incubation time on the current response, associated with the interaction of casein with *Leishmania* parasites, is shown on Figure 6. Hence, the current response decreased nonlinearly with the increase of the incubation time and reached a plateau after approximately 240 min, indicating the inhibition of the casein–*Leishmania* interaction. An incubation time of ~240 min seems to be appropriate for the effective inhibition of *Leishmanial* detection. Actually, it is not clear with which mechanism the antibiotic has made this inhibition, as revealed by chronoamperometric response decreases, e.g., either by blocking the active site of casein-GP63 interaction or simply through *Leishmania* lysis, which cause parasite number diminution. The experimental data are fitted with an exponential regression: (current density) = 4.2 × exp(−8×10^−2^ t), where the time t is expressed in minutes, with a correlation coefficient R^2^ = 0.9569.

## 4. Conclusions

In this work, AuNPs and casein@AuNPs conjugates were first synthesized, characterized by Transmission Electron Microscopy, Ultraviolet–Visible spectroscopy, and X-ray Photoelectron Spectroscopy, and used to design an electrochemical biosassay for *Leishmania* parasite detection and quantification by targeting its major surface protease GP63. The casein-conjugated gold nanoparticles are allowed to interact with *Leishmania* parasites taking advantage from the specificity of the interaction between casein and the GP63 proteins. This interaction was evaluated by chronoamperometry measurement of the current associated with AuNP catalysis of the hydrogen evolution reaction on screen-printed carbon electrodes. The assay was able to detect *Leishmania* with a detection limit of ~0.55 parasite/mL and over a linear range from 2 × 10^−2^ to 2 × 10^5^ parasites/mL, along with good reproducibility (RSD ≈ 4%). Briefly, it can be stated that the presented results are consistent with the development of a simple but highly accurate detection methodology of leishmania. The method could be promising for clinical application in human and veterinary medicine, especially in poor-resource settings. In this way, our future work will be devoted to the analysis of blood serums collected from both Leishmania-infected and noninfected dogs to assess the specificity and the sensitivity of the devised assay.
